# Neurotrophins regulate ApoER2 proteolysis through activation of the Trk signaling pathway

**DOI:** 10.1186/1471-2202-15-108

**Published:** 2014-09-19

**Authors:** Jorge A Larios, Ignacio Jausoro, Maria-Luisa Benitez, Francisca C Bronfman, Maria-Paz Marzolo

**Affiliations:** Departamento de Biología Celular y Molecular, Laboratorio de Tráfico Intracelular y Señalización, Facultad de Ciencias Biológicas, Pontificia Universidad Católica, Alameda 340, Santiago, 8320000 Chile; Millenium Nucleus in Regenerative Biology (MINREB), Pontificia Universidad Católica de Chile, Alameda 340, Santiago, 8320000 Chile; Departamento de Fisiología, Laboratorio de Neurobiología Celular y Regeneración. Facultad de Ciencias Biológicas, Pontificia Universidad Católica, Alameda 340, Santiago, 8320000 Chile; Department of Biochemistry, University of Geneva, Geneva, Switzerland

**Keywords:** ApoER2, Trk, p75^NTR^, Neurotrophins, Reelin, Proteolytic processing, Shedding, PC12 cells

## Abstract

**Background:**

ApoER2 and the neurotrophin receptors Trk and p75^NTR^ are expressed in the CNS and regulate key functional aspects of neurons, including development, survival, and neuronal function. It is known that both ApoER2 and p75^NTR^ are processed by metalloproteinases, followed by regulated intramembrane proteolysis. TrkA activation by nerve growth factor (NGF) increases the proteolytic processing of p75^NTR^ mediated by ADAM17. Reelin induces the sheeding of ApoER2 ectodomain depending on metalloproteinase activity. However, it is not known if there is a common regulation mechanism for processing these receptors.

**Results:**

We found that TrkA activation by NGF in PC12 cells induced ApoER2 processing, which was dependent on TrkA activation and metalloproteinases. NGF-induced ApoER2 proteolysis was independent of mitogen activated protein kinase activity and of phosphatidylinositol-3 kinase activity. In contrast, the basal proteolysis of ApoER2 increased when both kinases were pharmacologically inhibited. The ApoER2 ligand reelin regulated the proteolytic processing of its own receptor but not of p75^NTR^. Finally, in primary cortical neurons, which express both ApoER2 and TrkB, we found that the proteolysis of ApoER2 was also regulated by brain-derived growth factor (BDNF).

**Conclusions:**

Our results highlight a novel relationship between neurotrophins and the reelin-ApoER2 system, suggesting that these two pathways might be linked to regulate brain development, neuronal survival, and some pathological conditions.

## Background

The signaling of membrane surface receptors is regulated on a short-term basis by many different processes. Among these processes are posttranslational covalent modifications of the receptor’s intracellular region, internalization of the receptor and its ligand followed by lysosomal degradation, and/or proteolytic processing induced by soluble or membrane-integrated proteases. This latter process has been studied in different membrane-integrated receptors that have a single-pass transmembrane region, such as the amyloid precursor protein (APP), Notch receptor, p75^NTR^ neurotrophin receptor (p75^NTR^) and some members of the low density lipoprotein receptor family (LDLRs), including Apolipoprotein E receptor 2 (ApoER2), Megalin, and LRP1 [1–4]. The proteolytic processing of the receptors involves two general steps: 1) cleavage, called shedding, in the juxtamembrane extracellular domain, which results in the formation of a carboxy terminal membrane-integrated polypeptide (CTF, C-terminal fragment) and a soluble extracellular polypeptide (NTF, N-terminal soluble fragment), and 2) a second proteolysis event, carried out by the γ-secretase complex, at the transmembrane region of the membrane-integrated fragment, which produces two fragments, one of which corresponds to the intracellular domain (ICD) of the receptor [5]. In addition to regulating the half-life of the receptors, shedding and ICD production are processes that facilitate receptor signaling. For example, in the p75^NTR^ and Notch signaling pathways, the generation of proteolytic fragments is crucial for the correct signaling processes [6].

Many studies have focused on the mechanisms associated with the regulation of proteolytic processing of signaling receptors to understand the contribution of shedding to the intracellular signaling pathways. Neurotrophin receptors are among the principal study targets. The neurotrophins are trophic factors that act in the nervous system and regulate development and adult neuronal processes, such as neuronal survival or apoptosis [7], the outgrowth or withdrawal of neurites [8, 9], maturation and differentiation of sensory neurons [10], long-term depression (LTD) [11], and long-term potentiation (LTP) [12], among others [13]. Neurotrophin receptors include the Trk receptors (tyrosine kinase receptors). Binding of neurotrophins to a Trk receptor activates different signaling pathways, including the phosphatidylinositol 3-phosphate kinase (PI3K)/AKT pathway, the mitogen-activated protein kinase pathway (MAPK/ERK), and the phospholipase C-γ pathway [13]. Nerve growth factor (NGF) binds to TrkA [14], brain-derived neurotrophic factor (BDNF) binds to TrkB [15], and neurotrophin 3 (NT3) binds to TrkC [16].

The neurotrophin receptor p75^NTR^, a member of the tumor necrosis factor (TNF) receptor superfamily, binds to all the neurotrophins with the same affinity, unlike the Trk receptors [17]. p75^NTR^, along with its ligands, induces the regulation of intracellular pathways, including the activation of the c-Jun N-terminal kinase (JNK) pathway [18], the regulation of the NF-κB transcription factor [19, 20], and the modulation of RhoA GTPase activity [21, 22]. In the nervous system, p75^NTR^ has a role in neuronal apoptosis during nervous system development [23] and axon growth inhibition mediated by myelin and its interaction with the Nogo receptor, NgR1, and LINGO-1, which results in the activation of RhoA [24, 25]. Furthermore, when p75^NTR^ is co-expressed in neurons along with the Trk receptors, the apoptosis induced by p75^NTR^ signaling is abolished, and Trk receptor signaling is potentiated [26–29]. Many of the functions of p75^NTR^ in neurons are dependent on receptor proteolysis [30–39]. In PC12 cells, activation of TrkA by NGF induces the proteolytic processing of p75^NTR^, resulting in the accumulation of the p75^NTR^ CTF and also its ICD [40]. Furthermore, shedding of p75^NTR^ induced by NGF is mediated by the metalloprotease ADAM17 (a member of the disintegrin and metalloproteinase family), a process that facilitates NGF-TrkA signaling by stimulating the PI3K/AKT and MAPK/ERK pathways [37, 39]. Similarly, in cerebellar granule neurons, the expression of p75^NTR^ facilitates AKT activation in response to the activation of TrkB by BDNF [37]. Another signaling process of p75^NTR^ that is mediated by proteolytic processing of the receptor is the apoptosis of sensory neurons. In these neurons, activation of the apoptotic pathway requires the production of the p75^NTR^ ICD and the activation of the JNK pathway [38].

Another family of receptors expressed in the nervous system and related to brain development and adult neuronal functions is the LDLR family. Two receptors belonging to this family, ApoER2 and very low density lipoprotein receptor (VLDLR), together with their ligand reelin, are key components of the brain machinery involved in neuronal migration and positioning during brain development [41]. In this stage, reelin expressed by Cajal-Retzius cells [42, 43] facilitates the correct development of different regions of the CNS, including the hippocampus, cerebellum, and cerebral cortex [44–47]. In the adult stage, the functions of ApoER2 and its ligand reelin include the regulation of synaptic plasticity [47, 48], dendritic branching [49], actin remodeling [50], growth cone motility, filopodia formation [51], and neuronal survival [52, 53].

Reelin is an extracellular matrix protein that binds to ApoER2 and VLDLR, triggering the activation of several intracellular signaling pathways [54] including the interaction between the Dab1 adaptor with the cytoplasmic region of the receptors [55, 56]. Additionally, Dab1 is phosphorylated by the SRC family of kinases (SFK) [57–59], facilitating the activation of PI3K/AKT pathway [60]. PI3K regulates downstream effectors that are associated with cytoskeleton dynamics, such as cofilin [50, 61] and tau [62]. Like many surface receptors, ApoER2 signaling is regulated through lysosomal degradation after ligand binding and internalization [63]. Furthermore, as a result of the interaction between ApoER2 and its ligand, the receptor levels are downregulated by shedding [64], resulting in the production of the extracellular domain of the receptor and the carboxy-terminal fragment (ApoER2-CTF), which is the substrate of the γ-secretase complex. Other ligands of ApoER2, such as Apolipoprotein E, alpha-2 macroglobulin (α2M) and F-spondin, also induce the proteolysis of the receptor [64, 65]. However, there are few studies of the cellular mechanisms involved in the proteolysis of ApoER2 and the functional consequences that this process brings to the cells.

Both the reelin-ApoER2 and the neurotrophin signaling systems are expressed in neurons of the hippocampus, cerebellum, and cerebral cortex [13, 44–47] and share functions associated with brain development and adult neuronal functions, including participation in some pathological conditions such as schizophrenia [66]. Moreover, the signaling machineries associated with both types of receptors share intracellular signaling molecules with the PI3K/AKT pathway. Interestingly, there is little direct evidence of crosstalk between these two signaling receptor families. However, ligand binding to LRP1, a member of LDLR family, transactivates the Trk receptors through an SFK-dependent pathway in PC12 cells and neurons [67], demonstrating that apolipoprotein E-receptors have neurotrophic activity that is dependent on Trk receptor transactivation.

The aim of this study was to determine whether the activation of the Trk receptors by neurotrophins regulates shedding of ApoER2. We demonstrated that NGF regulates the proteolysis of ApoER2 in PC12 cells and that this process is dependent on TrkA signaling. In this model, we also demonstrated the participation of two signaling pathways in the constitutive shedding of ApoER2: the MAPK/ERK and PI3K/AKT pathways. In contrast, reelin was not able to induce shedding of p75^NTR^. Finally, modulation of ApoER2 proteolysis was also identified in primary cultures of cortical neurons, in which BDNF also induced the shedding of ApoER2.

## Results

### PC12 cells stably expressing ApoER2 respond to NGF and reelin

**Figure 1:**
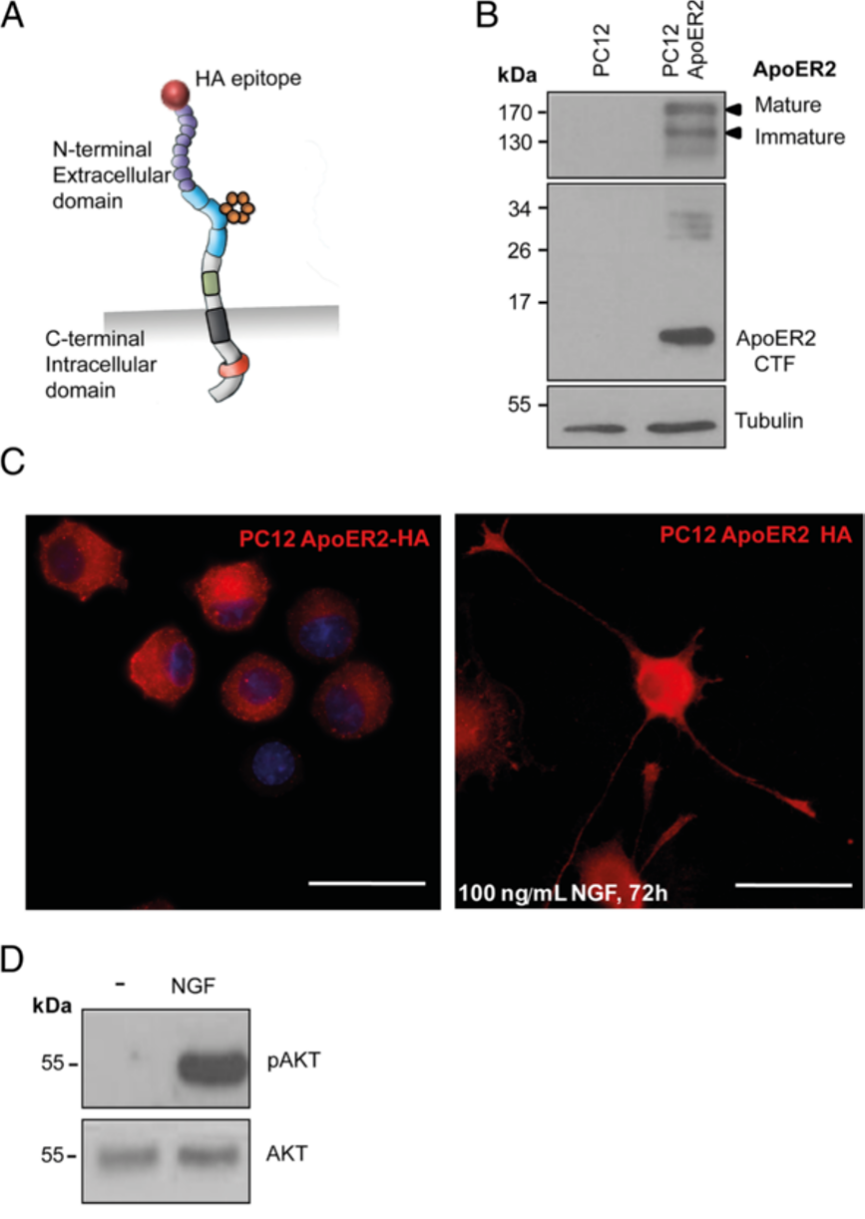
**Characterization of PC12 cells stably expressing HA-ApoER2. (A)** Schematic representation of the human HA-ApoER2 receptor transfected into PC12 cells. **(B)** Western blot showing the expression of HA-ApoER2 in stably transfected PC12 cells but not in wild type cells. ApoER2 was detected using an antibody that recognizes a region near the C-terminus of ApoER2. Both the mature (glycosylated) and immature forms (~170 and 130 kDa) of the receptor were detected. There are also recognized fragments close to 26–34 kDa and 17 kDa (corresponding to the receptor C-terminal fragment, CTF). α-tubulin is shown as a loading control. **(C)** Immunofluorescence of PC12 cells transfected with HA-ApoER2 (red) under basal conditions and after 72 h of NGF treatment (100 ng/mL) to induce differentiation. The cells expressed the receptor in different regions, including the plasma membrane and growth cones. Nuclear staining is shown in blue. Scale bar: 20 μm. **(D)** Western blots of cell lysates from PC12 cells stably expressing ApoER2. AKT phosphorylation was still detected after 2 h of incubation with NGF (100 ng/mL).

PC12 cells have been widely used to study the neurotrophin signaling pathways and their cellular functions. PC12 cells endogenously express TrkA and p75^NTR^ and, in response to the exogenous NGF, differentiate into a “neuronal cell phenotype” similar to sympathetic neurons found in the PNS [68, 69]. However, PC12 cells do not express high quantities of ApoER2. Therefore, to study the proteolysis of ApoER2, we generated stably transfected PC12 cells expressing the human full-length ApoER2 carrying an HA-tag at its N-terminus (HA-ApoER2) (Figure 1A). Western blot analysis using an antibody that recognizes the intracellular region of ApoER2 confirmed the expression of both the glycosylated (mature) and unglycosylated (immature) forms in cells transfected with the full-length receptor; additionally, some fragments of lower molecular weight, which represent proteolysis products, were also identified (Figure 1B). The receptor was localized throughout the cell, including the tips of the neurites in NGF-differentiated cells, as determined via detection of the HA epitope by immunofluorescence (Figure 1C). ApoER2 transfected cells also responded to NGF by activation of AKT (Figure 1D).

**Figure 2:**
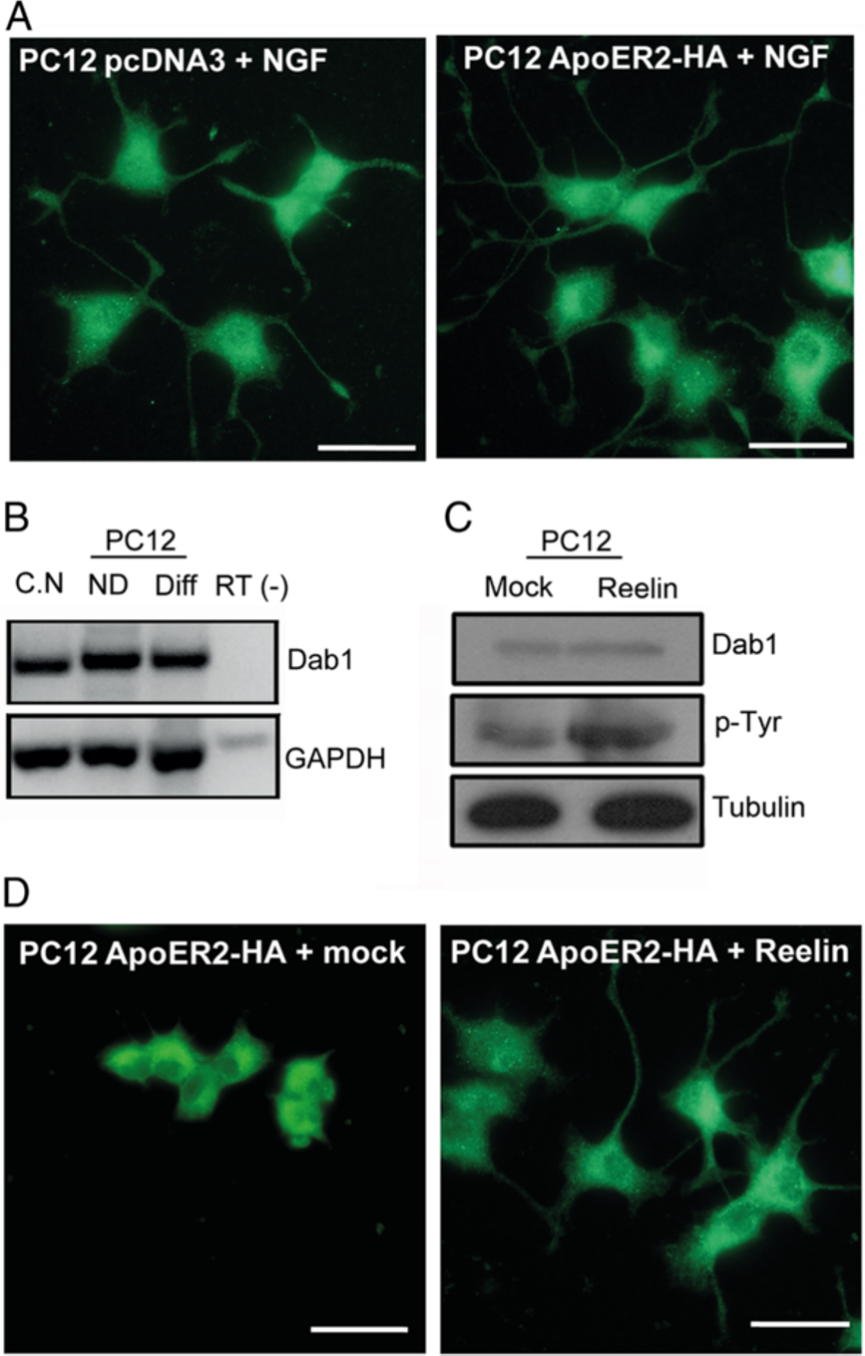
**ApoER2 expression does not affect PC12 differentiation or responses to neurotrophin. (A)** Differentiation of the PC12 control (pcDNA3) compared to cells stably expressing HA-ApoER2 with NGF (100 ng/mL) for 48 h. In both conditions, the cells were able to differentiate and extend neurites. **(B)** PCR showing the expression of Dab1 in total brain, embryonic cortical neurons, and both differentiated and undifferentiated PC12 cells stably expressing ApoER2. **(C)** PC12 expressing HA-ApoER2 cell were incubated with reelin-conditioned media or mock-conditioned media (control) for 30 min. Cells were lysed and the presence of total and phosphorylated Dab1 was detected by inmunoblot; α-tubulin is shown as a loading control. **(D)** The presence of ApoER2 in PC12 cells makes them responsive to reelin-conditioned media (48 h of treatment), as assessed by neurite extension.

PC12 cells that express HA-ApoER2 were activated by NGF in a similar way as the control cells (transfected with pcDNA3 plasmid) (Figure 2A). It is known that the ApoER2 ligand reelin induces neurite outgrowth in hippocampal and cortical neurons [70]. By PCR and by western blot, we confirmed the expression of the adaptor protein Dab1, which is required for the reelin canonical signaling pathway in PC12 cells (Figure 2B,C). In addition, we found that PC12 cells expressing ApoER2 respond to reelin; first, when they were treated with reelin-conditioned medium for 30 min it was possible to evidence the phosphorylation of Dab1 (Figure 2C) and second, the cells showed evident neurite extension after 48 h of incubation time with the ligand, (Figure 2D). Wild-type PC12 cells did not respond significantly to reelin (not shown), as expected from cells with low expression of endogenous ApoER2.

### NGF induces the proteolytic processing of ApoER2 in PC12 cells via TrkA

**Figure 3:**
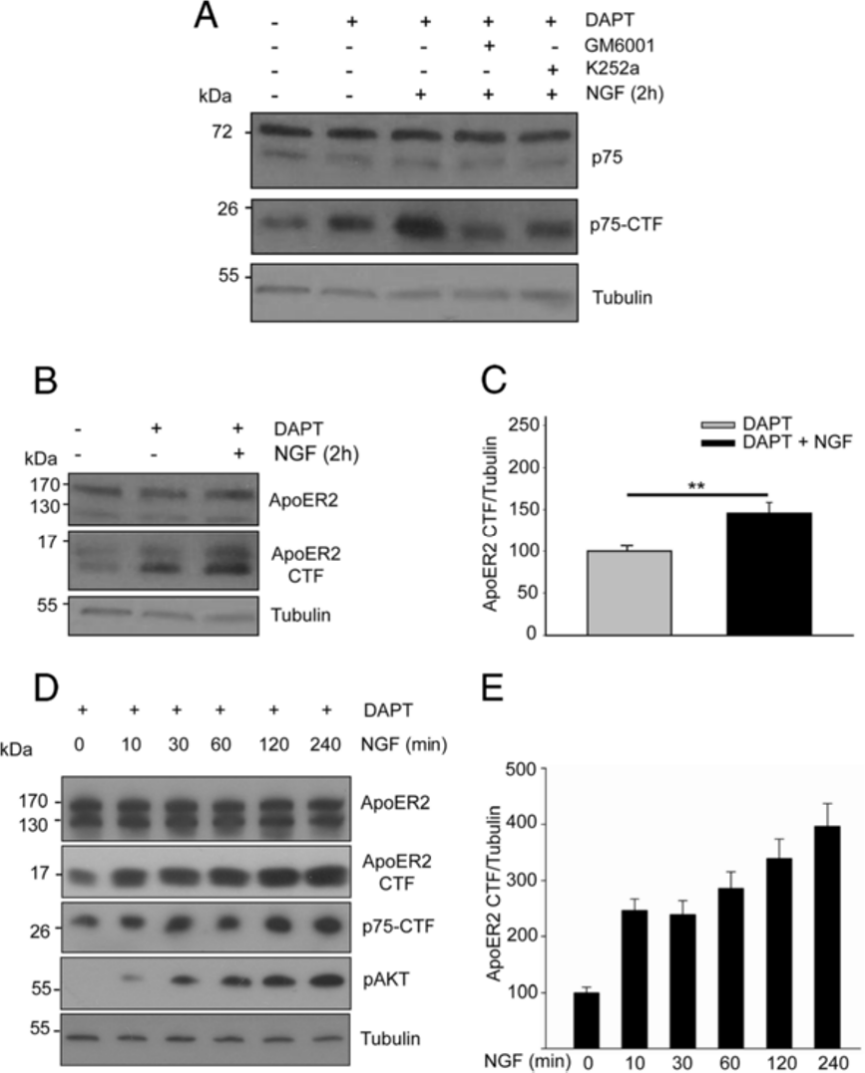
**NGF induces the proteolytic processing of ApoER2. (A)** Serum-starved PC12-ApoER2 cells were pre-treated with 10 μM DAPT (γ-secretase complex inhibitor), 50 μM GM6001 (metalloproteases inhibitor) and/or 100 nM K252a (Trk tyrosine kinase activity inhibitor) for 1 h and then incubated with 100 ng/mL NGF for 2 h. The blot shows full-length p75^NTR^, p75^NTR^ CTF, and α-tubulin as a loading control. As described [40], NGF induced the proteolysis of p75^NTR^ and, thus, the accumulation of the CTF. This process depends on TrkA tyrosine kinase activity and the metalloproteinases. **(B)** Cells were pre-treated with 10 μM DAPT for 1 h and then incubated with 100 ng/mL NGF for 2 h. ApoER2 and the proteolytic fragment ApoER2-CTF were recognized using antibodies against the intracellular region of the receptor. α-tubulin is shown as a loading control. **(C)** Quantification of blot levels of ApoER2-CTF normalized to the loading control α-tubulin and plotted as the average ± SD of four independent experiments. Student’s t-test, _**_P < 0.01. **(D)** PC12-ApoER2 was treated as previously described and then incubated with 100 ng/mL NGF for different times (0–240 min). Cell lysates were used for protein detection by western blot analysis. AKT phosphorylation is observed immediately after the addition of NGF. as well as the apparition of p75NTR CTF. α-tubulin is shown as a loading control. **(E)** Quantification of blot levels of ApoER2-CTF normalized to the loading control α-tubulin and plotted as the average ± SD of three independent experiments.

Wild type PC12 cells have been previously used to study the mechanisms involved in the regulation of p75^NTR^ proteolytic processing induced by NGF [40]. Therefore, we investigated whether this process operates in a similar way in the PC12 cells stably expressing HA-ApoER2. Cells were incubated with different pharmacological inhibitors, and the effects of the drugs on the proteolysis of p75NTF were analyzed. Inhibiting the γ-secretase complex DAPT, which has been used to study the proteolysis of APP, Notch, and ApoER2, among others substrates, induced the accumulation of the CTF of p75^NTR^, thus corroborating the participation of the γ-secretase complex in the proteolysis of p75^NTR^ (Figure 3A). Furthermore, incubating the cells with NGF for 2 hours induced an increase in the CTF levels, as shown previously, indicating that NGF regulates the proteolysis of p75^NTR^ in our cell system. The proteolysis of p75^NTR^ was prevented by pre-incubating the cells for 1 h with K252a, an inhibitor of Trk tyrosine kinase activity, thus supporting the role of TrkA in this process [40]. It is known that the shedding of p75^NTR^ induced by NGF is mediated by ADAM17 [39]. Accordingly, when cells were pre-treated with GM6001, a broad spectrum inhibitor of the proteases belonging to the metalloproteinases family, the effect of NGF on p75^NTR^ proteolysis was almost completely suppressed. These results show that PC12 cells stably expressing HA-ApoER2 respond to TrkA-NGF signaling and regulate p75^NTR^ proteolysis, just as the wild type PC12 cells [40].

PC12-ApoER2 cells that were incubated with DAPT accumulated the ApoER2 17 kDa CTF, as has been previously described in other cell types, which supports the constitutive proteolytic processing of ApoER2 [4, 64, 71] (Figure 3B). PC12-ApoER2 cells that were incubated with NGF for 2 h showed a significant increase in the CTF level (Figure 3B,C). Similarly, PC12-ApoER2 cells displayed a gradual increase in the CTF level when they were incubated for different times with the neurotrophin, which evident already from 10 minutes of treatment (Figure 3D). This observation indicates that the proteolytic processing of ApoER2 is regulated by NGF. As a positive control for NGF activity, we determined the proteolysis of p75^NTR^ and the phosphorylation of AKT.

**Figure 4:**
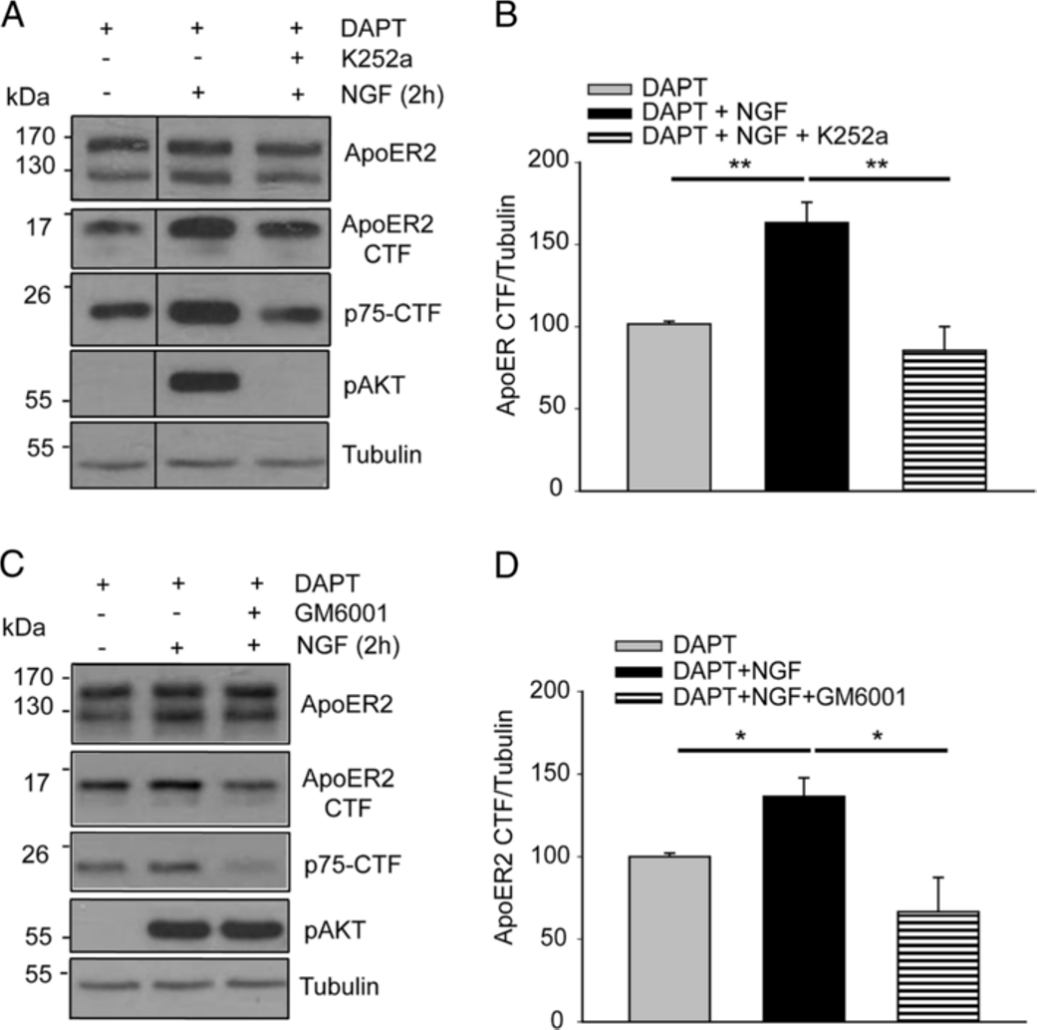
**ApoER2 proteolysis induced by NGF depends on TrkA tyrosine kinase activity and on metalloproteinase activity.** PC12-ApoER2 cells were serum-starved and pre-treated with 10 μM DAPT and **(A)** 100 nM K252a or **(C)** with 50 μM GM6001 for 1 h. Then, the cells were incubated with 100 ng/mL NGF for 2 h. ApoER2, ApoER2-CTF and the proteolytic fragment of p75^NTR^ (control) were determined by western blot analysis using antibodies directed against their intracellular regions. α-tubulin is shown as a loading control, and the phosphorylated form of AKT is a control for TrkA activation by NGF. **(B and D)** The levels of ApoER2 CTF were normalized to the loading control α-tubulin and plotted as the average ± SD of three independent experiments. One way ANOVA, Holm-Sidak post-hoc test, _**_P < 0.01; _*_P < 0.05.

The fact that NGF binds to the TrkA receptor as well as p75^NTR^ led us to investigate which of the receptors is involved in the regulated proteolysis of ApoER2 induced by NGF. Pre-treatment of PC12-ApoER2 cells with K252a abolished the AKT phosphorylation induced by NGF and also abrogated the increase in ApoER2-CTF levels induced by the neurotrophin (Figure 4A,B). This result demonstrates that the regulated proteolysis of ApoER2 depends on TrkA activity and not on p75^NTR^ signaling.

### Metalloproteinases of the ADAM family regulate the shedding of ApoER2 induced by NGF

Regulated proteolysis of many receptors, including p75^NTR^ and ApoER2, is achieved by metalloproteinases [64, 72, 73]. When PC12 cells were incubated with a general inhibitor of metalloproteinases of the ADAM family (GM6001) 1 h prior to the addition of NGF, receptor proteolysis was significantly decreased (Figure 4C,D). Therefore, this family of proteases is not only involved in p75^NTR^ shedding (Figure 3A) but also regulates the NGF-induced proteolytic processing of ApoER2.

### MAPK/ERK signaling pathway mediates p75^NTR^ but not ApoER2 proteolysis induced by NGF

**Figure 5:**
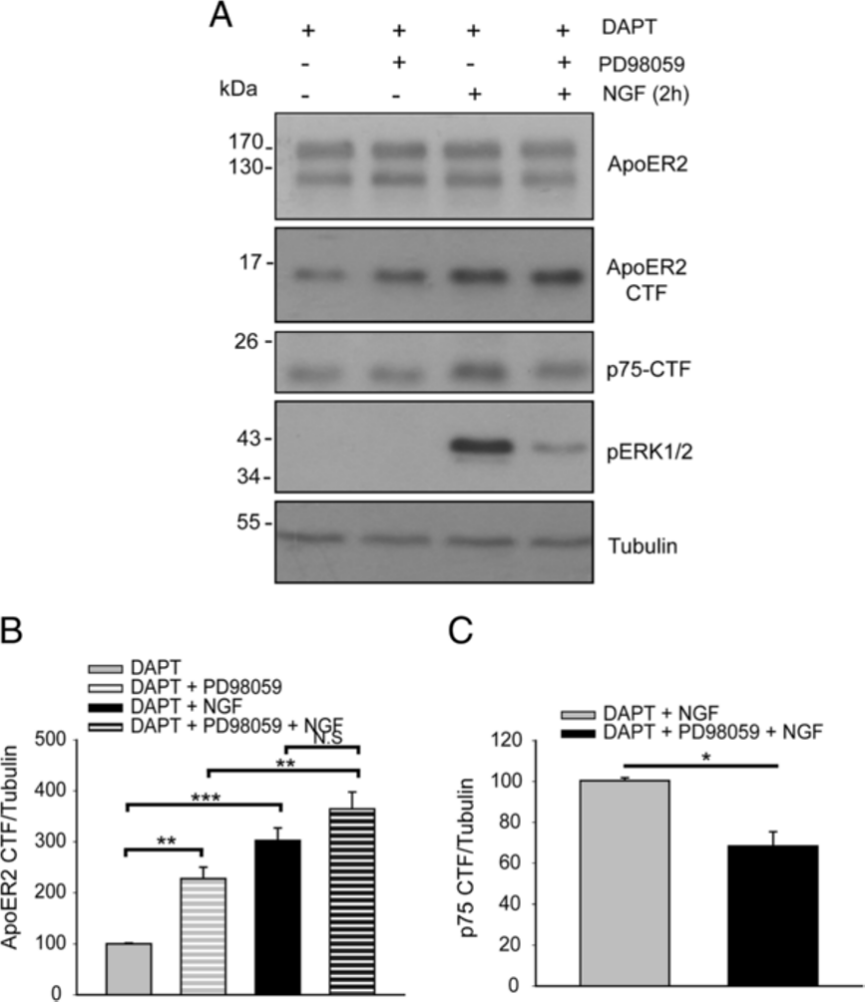
**MEK1/2 activity regulates the constitutive proteolysis of ApoER2 but is not involved in ApoER2 shedding induced by NGF.** **(A)** PC12-ApoER2 cells were serum-starved and pre-treated with 10 μM DAPT and 25 μM PD98059 for 1 h. Then, the cells were incubated with 100 ng/mL NGF for 2 h. ApoER2 and the proteolytic control p75^NTR^ were recognized by western blotting using antibodies directed against their intracellular regions. The activation of MEK1/2 by NGF was evidenced by recognition of phospho-ERK. α-tubulin is shown as a loading control. The blot levels of ApoER2 CTF **(B)** and p75^NTR^ CTF **(C)** were normalized to the loading control α-tubulin and plotted as the average ± SD of three independent experiments. One way ANOVA, Holm-Sidak post-hoc test, _*_P < 0.05, _**_P < 0.01, _***_P < 0.001, N.S, not significant.

Among the signaling pathways activated by NGF is the MAPK/ERK signaling pathway, which has been shown to regulate the proteolysis of p75^NTR^ [39]. Therefore, to further analyze the mechanisms involved in the NGF-induced proteolysis of ApoER2, PC12 cells were incubated with PD98059, an inhibitor of the ERK1/2 signaling pathway that controls MEK1/2 activity. Surprisingly, cells under this treatment displayed an increase in ApoER2 CTF levels, demonstrating that, under basal (no NGF) conditions, this MAPK/ERK signaling pathway downregulates the shedding of ApoER2 (Figure 5A,B). However, PC12 cells that were incubated with NGF in addition to the MEK1/2 inhibitor still showed an increase in the levels of ApoER2 CTF in response to the neurotrophin (Figure 5A,B) that was significantly higher than the CTF levels observed under basal MEK1/2 inhibition conditions. These results indicate that the MAPK/ERK signaling pathway is not involved in the proteolysis of ApoER2 induced by NGF. In contrast, the proteolysis of p75^NTR^ was dependent on the MAPK/ERK signaling pathway (Figure 5A,C). Therefore, although NGF stimulates the shedding of p75^NTR^ and ApoER2, the signaling pathways involved in the proteolytic processing of the two receptors are different.

### PI3K activity reduces ApoER2 basal proteolysis but is not required for the NGF-induced processing

**Figure 6:**
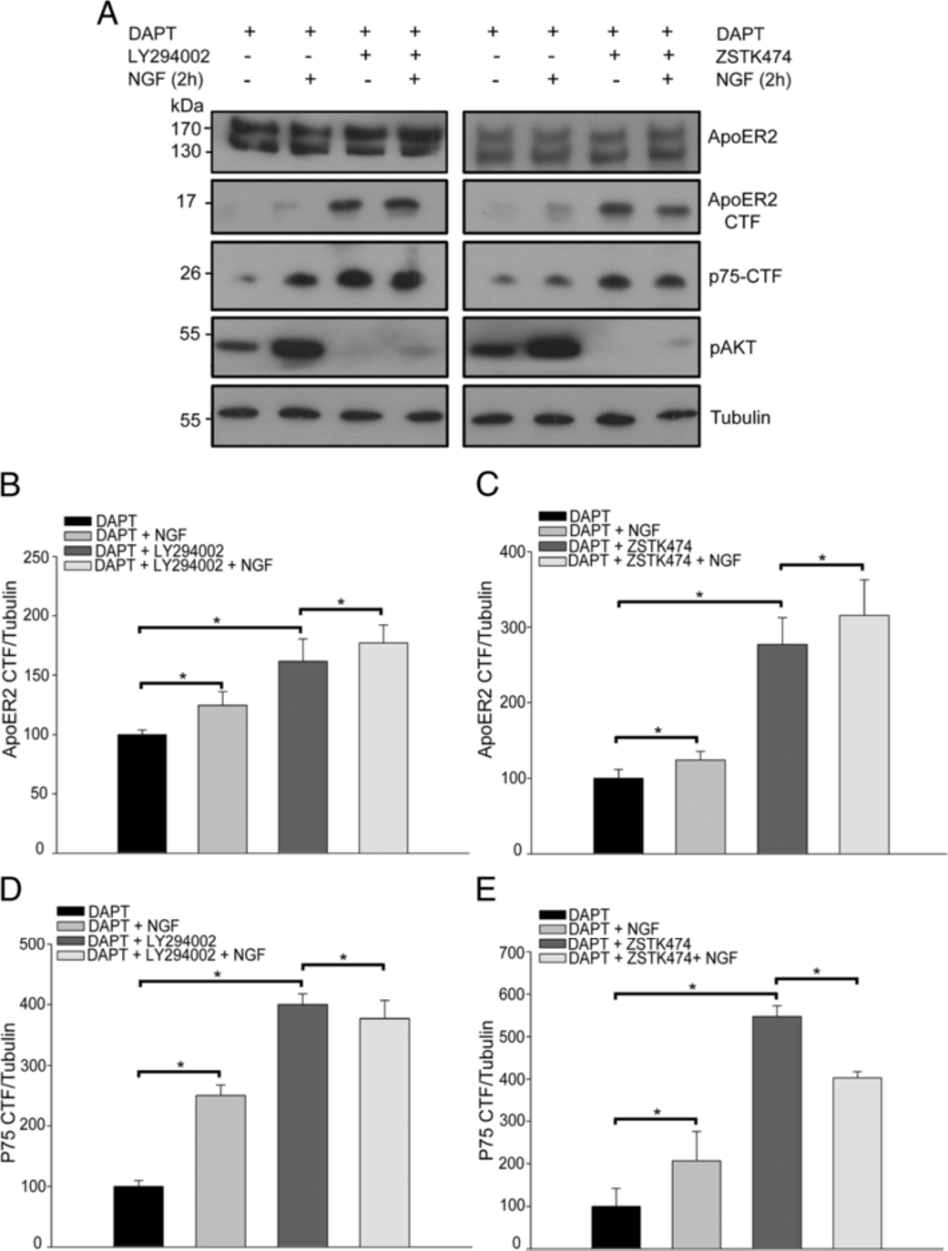
**PI3K activity regulates the constitutive levels of ApoER2 CTF but is not involved in ApoER2 shedding induced by NGF. (A)** PC12-ApoER2 cells were serum-starved and pre-treated with 10 μM DAPT and 50 μM LY294002 or 5 μM ZSTK474 for 1 h. Then, the cells were incubated with 100 ng/mL NGF for 2 h. ApoER2 and p75^NTR^ were recognized using antibodies directed against their intracellular regions. The activation of PI3K, induced by NGF, was determined by detection of phospho-AKT. α-tubulin is shown as a loading control. The blot levels of ApoER2 CTF **(B and C)** and of p75^NTR^ CTF **(D and E)** were normalized to the loading control α-tubulin and plotted as the average ± SD of three independent experiments. One way ANOVA, Holm-Sidak post-hoc test, _*_P < 0.01; N.S, not significant.

Another signaling pathway that is activated in response to NGF is the PI3K signaling pathway. This pathway is also activated by ApoER2 in response to its ligand reelin. Furthermore, this signaling pathway is known to mediate, among several effects, neuronal survival through the regulation of the apoptotic machinery [74]. To address the role of PI3K in the NGF-induced processing of ApoER2, PC12 cells were incubated with different PI3K inhibitors. Two pan-class I/II/III PI3K inhibitors , LY 294002 (reversal) and Wortmannin (covalent; not shown) as well as the class I/II PI3K inhibitor (ZSTK474), that does not affects class III PI3K (Vps34, involved in membrane trafficking in the endosomal route) [75, 76] were used and the ApoER2 and p75^NTR^ CTF levels were analysed by western blot (Figure 6A). Under basal conditions (no NGF) the CTF levels of both receptors were significantly increased following inhibitors treatment, indicating PI3K activity decreases the levels of the proteolytic fragments of the receptors (Figure 6B-E). Because all the inhibitors tested had the same effects, this suggests the possible involvement of class I/II and not class III PI3K. On the other hand, the NGF-induced processing of ApoER2 was not decreased when PI3K was inhibited; moreover the CTFs production was significantly increased under this condition, indicating that the machinery involved in the NGF-induced processing is not dependent on the activation of PI3K. In the same direction, the proteolysis of p75^NTR^ induced by NGF was not abolished, when PI3K activity was inhibited.

### ApoER2 proteolytic processing in cortical neurons

**Figure 7:**
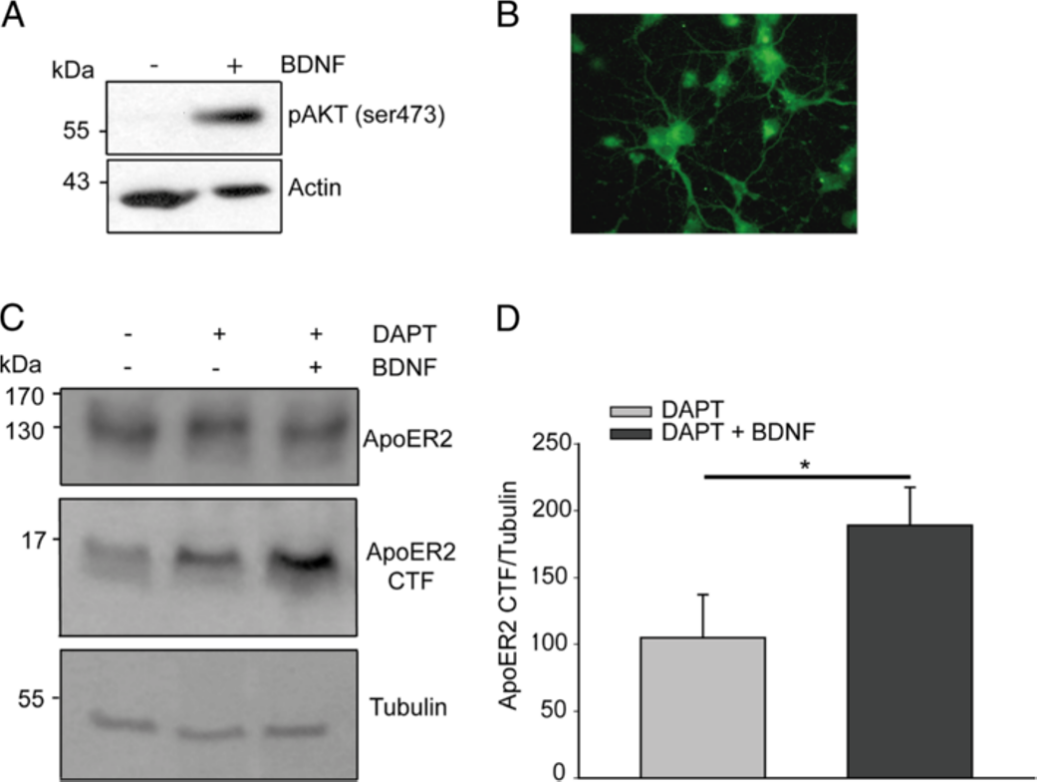
**BDNF induces the proteolytic processing of ApoER2 in cortical neurons. (A)** Cortical neurons (DIV 7) were treated with 100 ng/mL BDNF for 30 min. AKT phosphorylation levels shown are a result of TrkB activation by its ligand BDNF. Actin is shown as a loading control. **(B)** Immunofluorescence detection of ApoER2 in permeabilized cortical neurons from rat embryos using an antibody directed against the cytoplasmic domain of the receptor. **(C)** Serum-starved neurons were pre-treated with 10 μM DAPT for 1 h and then incubated with 100 ng/mL BDNF for 5 h. ApoER2 was recognized from cell lysates using an antibody directed against its intracellular pro-rich region. α-tubulin is shown as a loading control. **(D)** The blot levels of ApoER2 CTF were normalized to the loading control α-tubulin and plotted as the average ± SD of three independent experiments. Student’s t-test, _*_P < 0.05.

To further study the connection between ApoER2 and the neurotrophin system, we searched for potential cross-talk between the pathways in cultured rat embryonic cortical neurons that are known to express both ApoER2 and the TrkB receptor. Upon activation with BDNF, a TrkB ligand, cortical neurons responded with an increase in AKT phosphorylation (Figure 7A). ApoER2 was detected in these neurons by immunofluorescence (Figure 7B) and by western blot analysis as a full-length protein as well as its 17-kDa CTF (Figure 7C). Furthermore, this low molecular weight fragment accumulated after treatment with DAPT (Figure 7B,C), which indicates that the γ-secretase complex, as expected, participates in the basal processing of ApoER2-CTF in cortical neurons. When DAPT-pre-treated neurons were subsequently incubated with BDNF for 5 h, the levels of the ApoER2 CTF significantly increased (Figure 7C,D), thus strengthening the data obtained in PC12 cells expressing HA-ApoER2 regarding the role of neurotrophins in receptor shedding.

### Reelin regulates ApoER2 degradation and proteolysis but not of p75

**Figure 8:**
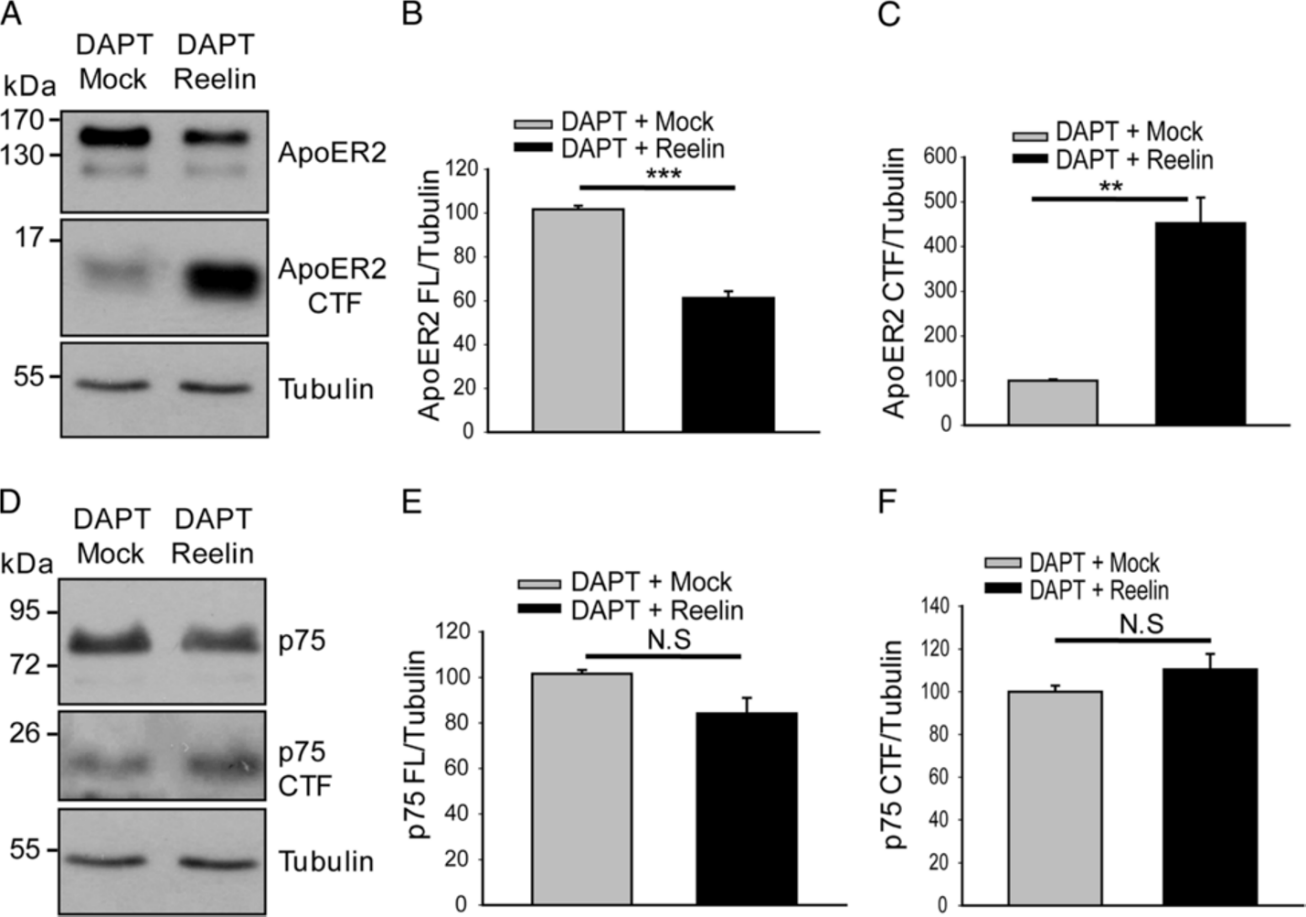
**Reelin induces ApoER2 proteolysis in PC12 cells without affecting p75 CTF levels.** Western blot of cell lysates from PC12 cells stably expressing HA-ApoER2. Serum-starved cells were pre-treated with 10 μM DAPT and then incubated with recombinant reelin or the control medium (mock) for 2 h. ApoER2 **(A)** and p75 **(D)** were recognized using an antibody directed against its intracellular region. α-tubulin is shown as a loading control. **(B and C)** Quantification of blot levels of ApoER2 and its CTF; both quantifications were normalized to the loading control α-tubulin and plotted as the average ± SD of three independent experiments. Student’s t-test, _**_P < 0.005, _***_P < 0.001. **(E and F)** Quantification of the blot levels of p75 and its CTF; both quantifications were normalized to the loading control α-tubulin and plotted as the average ± SD of three independent experiments. Student’s t-test, not significant (N.S).

Reelin downregulates the protein levels of ApoER2, inducing both proteolytic processing [64] and lysosomal degradation [63]. When PC12 HA-ApoER2 cells were treated with reelin, the levels of the mature form of the receptor decreased (Figure 8A,B). Moreover, reelin induced the accumulation of the CTF of ApoER2, thus corroborating the role of this ligand in the proteolysis of ApoER2 (Figure 8A,C). To investigate crosstalk between the neurotrophin family and the reelin-ApoER2 system, we analyzed the levels of full-length p75^NTR^ and the CTF of p75^NTR^ after treatment with reelin for 2 h. The results indicated that, under this experimental condition, reelin does not affect the normal degradation of p75^NTR^ (Figure 8D,E,F ).

## Discussion

The nervous system works as a complex network in which the neurons are the primary components. Many studies have shown the involvement of diverse signaling pathways both in the development of the nervous system and during adult stages. For example, the expression of neurotrophins and their receptors in neurons are crucial for the correct functioning of the CNS and PNS [9]. Similarly, reelin and its receptor ApoER2 participate in neuronal processes during development and in adulthood [41, 48, 70, 77, 78]. However, despite the large number of studies that have been conducted in neurons and the discovery of an enormous number of proteins involved in signaling, less is known about the connections between the different signaling systems functioning in the brain.

Based on reports showing that the neurotrophin and reelin signaling pathways share common intracellular effectors and that the receptors of both families are regulated by proteolytic processing, we studied whether crosstalk may exist between these two systems at the level of the regulation of proteolysis. As it was reported previously, p75^NTR^ proteolysis is regulated by the activation of TrkA receptor. Until now, there was no evidence of the regulation of other proteolytic processes involving Trk activation. In this work, we found that the activation of TrkA by NGF induced the proteolysis of ApoER2, specifically affecting the shedding of the receptor and resulting in the accumulation of its CTF. These results demonstrate a connection between the neurotrophin system and the reelin pathway. Similar to the shedding of p75^NTR^ the TrkA-regulated proteolysis of ApoER2 was dependent on the metalloproteinases of the ADAM family. Although we did not identify the metalloproteinase involved in the shedding of ApoER2, the participation of the membrane integrated metalloproteinase ADAM17 has been described in the proteolysis of p75^NTR^ induced by NGF [39].

The shedding of p75^NTR^ induced by NGF in PC12 cells is dependent on MEK1/2 activity [39] and our data supported this finding. The activation of the MAPK/ERK pathway promotes the phosphorylation of ADAM17 [79]. The phosphorylation of ADAM17 in the intracellular region (threonine 735) is known to affect its intracellular localization, increase its concentration at the cell surface, and facilitate its maturation [79]. Moreover, the proteolysis of p75^NTR^ induced by NGF is dependent on the phosphorylation of threonine 735 of ADAM17 [39]. However, while Kommaddi et al. showed that NGF stimulates ADAM17 phosphorylation, they did not observe a change in the surface levels of the metalloproteinase. Furthermore, activation of this signaling pathway is known not only to modulate the surface levels of the metalloproteinase but also to shift the balance from ADAM17 dimers to monomers. This process stimulates the dissociation of tissue inhibitor of metalloproteinase-3 (TIMP3) from ADAM17, thus activating the metalloproteinase [80]. Previous studies have shown that TIMP3 decreases the level of ApoER2-soluble extracellular fragments and CTFs, suggesting an active role for α-secretase in the first step of ApoER2 processing at the plasma membrane [73]. In contrast, ApoER2 proteolysis induced by NGF was not dependent on MEK/ERK activation, which is the first difference between the regulated proteolysis of ApoER2 and p75^NTR^. This result suggests that the proteolysis of ApoER2 induced by NGF may be mediated by metalloproteinases other than ADAM17. Surprisingly, under basal conditions, our results show that MEK activity decreases the levels of the ApoER2 CTF, contrary to what is known about the regulation of ADAM17 activity on different substrates [72, 81]. This result therefore strengthens the idea that other metalloproteinases participate in the shedding of ApoER2 under these conditions.

The PI3K/AKT pathway in neurons is another signaling pathway that is activated in response to NGF, and its activation is dependent on TrkA activity. The PI3-kinases catalyze the phosphorylation of the phosphatidylinositols (PtdIns), a family of minority lipids present in the cytosolic side of cell membranes. Many of the products of PI3K (PtdIns3P, PtdIns(3,4)P2, PtdIns(3,5)P2, and PtdIns(3,4,5)P3) function in specific regions of the cell to recruit various proteins involved in protein trafficking and signaling [82, 83]. Our results demonstrate that inhibiting the basal activity of PI3K via the pan-class I/II/III PI3K inhibitor LY 294002 as well as the class I/II PI3K inhibitor ZSTK474 [75, 76] under a condition in which the activity of the γ-secretase complex is inhibited with DAPT, results in a significant accumulation of the proteolytic fragments of ApoER2. Many studies have shown the importance of PI3K in the trafficking of cell surface receptors through the endocytic pathway. For example, the transferrin receptor and receptors belonging to the LDLR family (LDLR, LRP1 and VLDLR) display impaired trafficking via the endocytic compartments after treatment with wortmannin [84, 85] a covalent pan-class I/II/III PI3K inhibitor. Specifically, the receptors accumulate in early endosomes and are prevented from progressing to late endosomes, which affects their lysosomal degradation, and also display impaired recycling to the cell surface. Therefore, the observed increase in the levels the CTF of ApoER2 (and also of p75^NTR^) when PI3K activity is inhibited could be due to an increase in receptor shedding and/or reduced CTF degradation, by a γ-secretase independent pathway. Similarly to what we found for MEK/ERK activation, ApoER2 proteolysis induced by NGF was not dependent on PI3K activation. Thus, we suggest that the NGF-regulated proteolysis of ApoER2 does not depend on PI3K activity, in the same way of what was observed for p75^NTR^.

Our results using cultures of primary cortical neurons demonstrate a connection between neurotrophin signaling and the proteolysis of endogenous ApoER2. The levels of ApoER2-CTF were regulated by BDNF, thus reinforcing the results obtained in PC12 cells. Our data, together with previous studies in neurons that show common functions and intracellular pathways between the neurotrophin and reelin-ApoER2 systems, suggest that these two families may be coordinated to regulate neuronal functions both during development and under different physiological conditions in the adult brain. A recent report indicated a genetic interaction between reelin and BDNF that is dependent on the sex hormones [86]. Moreover, the actions of BDNF in Cajal-Retzius cells during development decrease the expression of reelin [87], while the mRNA expression of VLDLR, the other reelin receptor, increases through TrkB [88]. Furthermore the Reeler mouse, which lacks reelin, has significantly decreased BDNF levels along with lower TrkB activity [89]. In addition, mice devoid of BDNF during development have significantly increased reelin levels [87]. Interestingly, the intracellular fragment of ApoER2 generated by the activity of the γ-secretase complex, the ApoER2-ICD, can inhibit the expression of reelin at the transcriptional level [90]. Therefore, the documented reduction of reelin induced by BDNF [87] could be dependent, at least in part, on the neurotrophin- induced proteolysis of ApoER2, as reported in our work. Overall, these data suggest a functional relationship between these two systems that may be relevant for pathological conditions such as schizophrenia [91–95] and autism [96–98].

ApoER2 is an endocytic receptor that follows the degradative pathway to the lysosomes in response to reelin binding [63]. Reelin also induces the proteolytic processing of the receptor by a still unknown mechanism [4, 64]. When PC12 cells were treated with reelin and DAPT, the levels of mature ApoER2 decreased, and the CTF of the receptor accumulated. Therefore, in our cell model, receptor degradation is stimulated by its ligand. Reelin was also able to induce neurite outgrowth in PC12 cells expressing ApoER2, but it did not affect the levels of p75^NTR^ or its CTF. These results indicate that, in contrast to the effect of NGF on ApoER2 processing, reelin signaling does not regulate the functionality of p75^NTR^ or the formation of its fragments. However, we could not discount that the processing of p75^NTR^ could be affected by reelin in a different cellular context because the levels of p75^NTR^ in our cortical neurons were too low to be detected as proteolytic fragments.

To date, little is known about the function of the proteolytic fragments of ApoER2. There are studies showing that the extracellular fragment of ApoER2 produced after shedding have the ability to interact with ligands found in the extracellular medium [99]. Additionally, the ApoER2-ICD can translocate to the nucleus [100] where, as mentioned before, it may be able to inhibit reelin transcription [90].

## Conclusions

Even though the signaling pathway regulating ApoER2 shedding, induced by neurotrophins, was different than the one described for p75^NTR^ in terms of the participation of MEK1/2 activity, our study clearly gives new insights into the crosstalk between neurotrophins and the reelin receptors, specifically through the activation of ApoER2 shedding by the Trk receptors. In contrast, the ApoER2/reelin pathway would not regulate p75^NTR^ in terms in its proteolytic processing.

Further studies are required to understand the cellular role of the neurotrophin-induced ApoER2 processing in neurons. Plausible functions of ApoER2 processing, induced by its own ligand and/or by neurotrophin signaling, could be involved in fine-tuning the regulation of the reelin signaling cascade that should occur at the synapse and/or during neuronal migration.

## Methods

### Antibodies

We used a rabbit polyclonal antibody directed against a region near the C-terminal of ApoER2 (A3481, Sigma). The rabbit polyclonal antiserum against the recombinant human ApoER2 cytoplasmic domain and the mouse monoclonal anti-HA have been described before [101]. We also used a p75^NTR^ rabbit polyclonal antibody (07–476, Millipore), a mouse monoclonal anti-β-tubulin antibody (05–661, Millipore), a mouse monoclonal anti-actin antibody (MAB1501R, Chemicon), a rabbit polyclonal anti-AKT antibody (#9272, Cell Signaling), a rabbit monoclonal anti-phosphorylated AKT antibody (#4060, Cell Signaling), a mouse monoclonal anti-phosphorylated ERK antibody (sc-7383, Santa Cruz Biotechnology), a rabbit polyclonal anti-Dab1 antibody (AB5840, Chemicon International), and a mouse monoclonal anti-MAP2 antibody (MAB378, Chemicon). We used horseradish peroxidase (HRP)-conjugated secondary antibodies (Chemicon) and Alexa 555- and 488-conjugated goat anti-mouse and anti-rabbit secondary antibodies (Molecular Probes).

### Plasmids

The N-terminally HA-tagged full-length ApoER2 in the pCDNA3 vector [GenBank: NM_004631] was described previously [101, 102].

### Cell lines and culture conditions

PC12 cells were maintained in DMEM-high glucose (Invitrogen) with 6% fetal bovine serum [FBS, (Hyclone)], 6% horse serum [HS, (Hyclone)], 100 U/mL penicillin and 100 μg/mL streptomycin in a 5% CO_2_ incubator at 37°C. PC12 cells were stably transfected with Lipofectamine 2000 reagent (Invitrogen) according to the manufacturer’s protocol. The cells were selected with 0.8 mg/mL Geneticin [G418, (Hyclone)] and maintained with 0.4 mg/mL G418. HEK-293 cell lines stably expressing reelin or the control vector pcDNA3 were grown in DMEM-high glucose supplemented with 10% FBS, 100 U/mL penicillin, and 100 μg/mL streptomycin and maintained with 0.4 mg/mL G418 in a 5% CO_2_ incubator at 37°C.

### Primary culture of rat embryonic cortical neurons

The protocols to obtain neurons from rat (Sprague–Dawley) embryos were performed with approval from the Bioethical Board for animal studies at the Facultad de Ciencias Biológicas and according to the Guide for the Care and Use of Laboratory Animals of CONICYT. Cortical neurons were prepared and cultured essentially as described [71]. Cerebral cortexes from 18-day-old embryos were washed two times with cold Hank’s medium and digested with Hank’s 0.5% trypsin-EDTA (Invitrogen) for 18 min at 37°C. The tissue was resuspended in DMEM-high glucose supplemented with 10% FBS, 100 U/mL penicillin and 100 μg/mL streptomycin. Cells were counted and plated (200,000 cells/cm^2^) in poly-L-lysine (mol wt 30,000-70,000, Sigma) pre-treated Petri dishes and incubated in a 5% CO_2_ incubator at 37°C. After four hours, cell medium was replaced with a neuronal maintenance medium comprised of Neurobasal medium (Invitrogen), 1X B27 supplement (Invitrogen), 2 mM L-Glutamine (Invitrogen), 100 U/mL penicillin and 100 μg/mL streptomycin. After 24 h, the neurons were treated with 1 μM arabinofuranosyl cytidine (Ara-C, from Sigma).

### Reelin conditioned medium

HEK-293 cells stably expressing reelin or the control vector pcDNA3 were the kind gift of Dr. Tom Curran (University of Pennsylvania, USA). The cells were grown in 10-cm plates until they reached 80% confluence in a 5% CO_2_ incubator at 37°C. They were washed once with PBS and incubated for 24 h with serum-free medium (DMEM-high glucose, 100 U/mL penicillin and 100 μg/mL streptomycin) in a 5% CO_2_ incubator at 37°C. Later, the cell medium was collected and centrifuged at 1,000 rpm for 5 min, and the supernatant was stored at 4°C. The remaining plates with cells were filled again with serum-free medium and incubated for 24 h in a 5% CO_2_ incubator at 37°C. The collecting procedure was repeated a total of three times (72 h), and then the supernatant was concentrated 10 times using Amicon Ultra-15 centrifugal filter units with a 100 kDa membrane cut-off, according to the manufacturer’s protocol (Millipore).

### Cell treatments and Western blot analysis

PC12 cells stably expressing HA-ApoER2 were grown in 6-well plates coated with poly-L-lysine. When the cells were 80% confluent, they were washed once with warm PBS and incubated with serum-free medium (DMEM high glucose) for 2 h in at 37°C. After serum deprivation, the cell medium was replaced with fresh serum-free medium, and the corresponding inhibitory drugs were added for 1 h at 37°C. For inhibitory agents, we used 100 nM K252a (Alomone Labs), 10 μM DAPT, 25 μM PD98058, 50 μM GM6001 100 nM wortmannin and 50 μM of LY294002 (all from Calbiochem) and 5 μM of ZSTK474 (Selleck Chemicals LLC) Later, the cells were treated with 100 ng/mL NGF (Alomone Lab) for different times in a 5% CO_2_ incubator at 37°C. To treat the PC12 cells with reelin, the cells were incubated with either the ligand-enriched or the control medium (mock) for 2 h in a 5% CO_2_ incubator at 37°C. The cells were lysed with lysis buffer (20 mM Tris, 150 mM NaCl, 1% NP-40, 10% glycerol, 2 mM EDTA, 1 mM PMSF, 4.7 μM leupeptin, 1 μM pepstatin, 1 μM antipain, 1 μM aprotinin, 1.5 μM benzamidine, 1 mM sodium orthovanadate, 5 mM NaF, 1 mM glycerol phosphate) and centrifuged at 14,000 rpm for 5 min at 4°C, and the protein concentration of the supernatant was quantified with the BCA protein assay kit according to the manufacturer’s protocol (Pierce). Samples were denatured in denaturing buffer (0.3 M Tris, 0.35 M SDS, 50% glycerol, 0.05% blue bromophenol, and 25% β-mercaptoethanol) by boiling for 5 min. The proteins were subjected to SDS-PAGE under reducing conditions, transferred to a polyvinylidene difluoride (PVDF) membrane, and incubated with a blocking solution (0.1% Tween-20, 5% nonfat powdered milk, and PBS) for 30 min at room temperature. Later, the corresponding primary antibody was added (anti-tubulin 1:10,000; anti-ApoER2 A3481 1:10,000; anti-p75^NTR^ 1:1,000; anti-p-ERK 1:1,000; anti-AKT 1:1,000; anti-p-AKT 1:1,000; anti-Dab1 1:1,000 and 1:1000 anti-p-Tyr) in blocking solution for 16 h at 4°C. The PVDF membranes were washed with the blocking solution three times and incubated with HRP-conjugated antibodies (1:5,000) for 2 h at room temperature in blocking solution. Then, the immunoreactive proteins were detected using the ECL system according to the manufacturer’s protocol (Pierce).

Rat cortical neurons were grown in 6-well plates for 7 DIV, washed once with warm serum-free medium (Neurobasal), and incubated with the same medium for 1 h in a 5% CO_2_ incubator at 37°C. After serum deprivation, the cell medium was replaced with fresh serum-free medium and the corresponding inhibitory drug, similar to the PC12 cell treatment described earlier. The neurons were incubated for 1 h in a 5% CO_2_ incubator at 37°C and were later treated with 100 ng/mL BDNF (Alomone Labs, Jerusalem, Israel) for different times in a 5% CO_2_ incubator at 37°C. The cell lysis protocol and SDS-PAGE were performed as described for the PC12 cells.

### Determination of Dab1 mRNA expression

Total RNA was extracted using the RNA-Solv® Reagent (Omega Biotec). The extracted RNA was quantified by spectrophotometry at 260-nm optical density in a NanoDrop (ND-1000) Spectrophotometer (NanoDrop Technologies, Rockland, DE). For RT-PCR, first-strand synthesis was performed with the M-MLV reverse transcriptase (Thermo Scientific Inc.*)* In brief, 1 μg of total RNA was incubated with DNase I for 15 min at room temperature. Then, 1 μL of EDTA was added, and the reaction was incubated 10 min at 65°C. Finally, 1 μL of random primers were added, and the reaction was incubated at 70°C for 5 min. After incubation, dNTPs, 10× PCR Buffer, RNase inhibitor, and reverse transcriptase were added, and the reaction was incubated at 25°C for 5 min followed by 25°C for 10 min, 42°C for 60 min, and 70°C for 10 min. The resulting cDNA was used for Dab1 PCR. The primers for Dab1 amplification were designed for optimal performance using the OligoAnalyzer 3.1 of the IDT Integrated DNA Technologies and Net primer free software from PREMIER Biosoft International (forward CATTGCGAAGGACATCACAG; reverse CGGCTTCACACTGCTTA). The cycling conditions for the amplified products were as follow: 95°C for 0.45 seconds, 50°C for 1 min, 72°C for 0.45 seconds (35 cycles). The amplified products were run on a 1% gel, and the bands were visualized under UV light after staining with Red Gel (Thermo Scientific Inc.).

### Immunofluorescence

PC12 cells stably expressing HA-ApoER2 were plated on glass coverslips coated with poly-L-lysine. The cells were washed with PBS and fixed with 3% paraformaldehyde solution (3% PFA, 4% sucrose and PBS) at room temperature for 15 min. After three washes with PBS for 5 min each, the cells were permeabilized with 0.2% Triton X-100 in PBS for 10 min and then washed three times with PBS. Coverslips were incubated at room temperature with a blocking solution (0.2% gelatin from bovine skin (Sigma) and PBS) for 1 h. Later, the cells were incubated with a mouse anti-HA antibody diluted in blocking buffer at 4°C overnight. The coverslips were washed three times with PBS and then incubated with Alexa 555-conjugated anti-mouse antibody for 30 min at 37°C. After three washes with PBS, the coverslips were mounted with Fluoromount mounting medium (Sigma) on glass slides.

The immunofluorescence protocol for cortical neurons was the same as that used for the PC12 cells, but a different blocking buffer [5% gelatin from cold water fish skin (Sigma) and PBS] was used. Neurons were incubated with the anti-ApoER2 cytoplasmic domain antibody (1:1,000) in blocking buffer overnight at 4°C. Coverslips were washed three times with PBS and then incubated with Alexa 555-conjugated anti-mouse antibody and Alexa 488-conjugated anti-rabbit antibody for 30 min at 37°C. After three washes with PBS, the coverslips were mounted with Fluoromount mounting medium on glass slides.

### Statistical analysis

Quantification of the blots was performed with the ImageJ 1.45 s software. Statistical analysis and graphing were performed with SigmaPlot 11.0 using Student’s t-test or one way ANOVA with the Holm-Sidak post-hoc test, depending on the experiment.

## Abbreviations

ADAM17: A Disintegrin and metalloproteinase 17
ApoER2: Apolipoprotein E receptor 2
APP: Amyloid precursor protein
BDNF: Brain-derived neurotrophic factor
CTF: C-terminal fragment
ICD: Intracellular domain
JNK: c-Jun N-terminal kinase
LTD: Long term depression
LTP: Long-term potentiation
LDLR: Low density lipoprotein receptor
MAPK: Mitogen-activated protein kinase
MEK: Mitogen-activated protein kinase
NGF: Nerve growth factor
NTF: N-terminal fragment
NT3: Neurotrophin 3
PC12: Rat pheochromocytoma cell line
PI3K: Phosphatidylinositol 3 kinase
PtdIns: Phosphatidylinositols
p75NTR: Neurotrophin receptor
SFK: Src family kinases
TIMP3: Tissue inhibitor of metalloproteinase-3
VLDLR: Very low density lipoprotein receptor.
